# Progressive Intracranial Vertebral Artery Dissection Presenting with Isolated Trigeminal Neuralgia-Like Facial Pain

**DOI:** 10.1155/2015/387139

**Published:** 2015-06-03

**Authors:** Tomoki Nakamizo, Takashi Koide, Hiromichi Miyazaki

**Affiliations:** ^1^Department of Neurology, Hiratsuka City Hospital, 1-19-1 Minamihara, Hiratsuka, Kanagawa 254-0065, Japan; ^2^Department of Neurosurgery, Hiratsuka City Hospital, 1-19-1 Minamihara, Hiratsuka, Kanagawa 254-0065, Japan

## Abstract

Intracranial vertebral artery dissection (IVAD) is a potentially life-threatening disease, which usually presents with ischemic stroke or subarachnoid hemorrhage. IVAD presenting with isolated facial pain is rare, and no case with isolated trigeminal neuralgia- (TN-) like facial pain has been reported. Here, we report the case of a 57-year-old male with IVAD who presented with acute isolated TN-like facial pain that extended from his left cheek to his left forehead and auricle. He felt a brief stabbing pain when his face was touched in the territory of the first and second divisions of the left trigeminal nerve. There were no other neurological signs. Magnetic resonance imaging (MRI) of the brain 7 days after onset revealed dissection of the left intracranial vertebral artery without brain infarction. The pain gradually disappeared in approximately 6 weeks, and the patient remained asymptomatic thereafter, except for a brief episode of vertigo. Follow-up MRI revealed progressive narrowing of the artery without brain infarction. This case indicates that IVAD can present with isolated facial pain that mimics TN. IVAD should be considered in the differential diagnosis of acute facial pain or TN.

## 1. Introduction

Intracranial vertebral artery dissection (IVAD) is a potentially life-threatening disease, which usually presents with ischemic stroke or subarachnoid hemorrhage (SAH) [1]. Headache often accompanies IVAD [1], and infrequently, it is the only symptom [1, 2]. However, IVAD presenting with isolated facial pain is rare; only 1 case has been reported [3]. Furthermore, no case of IVAD presenting with trigeminal neuralgia- (TN-) like facial pain has been reported. Here, we present the case of a patient with IVAD who presented with isolated facial pain mimicking TN.

## 2. Case Presentation

A 57-year-old man had been healthy until 7 days earlier when he suddenly noticed left facial pain. He visited a hospital where he underwent computed tomography of the head, which showed normal findings. The pain continued despite treatment with carbamazepine; thus, he was admitted to our hospital.

His left facial pain extended from the left cheek to the left forehead and auricle. On examination, the blood pressure was 156/108 mmHg and pulse rate was at 76 beats/min. He was alert and well oriented. He felt a brief stabbing pain when his face was touched in the territory of the first and second divisions of the left trigeminal nerve. There was no ptosis. His pupils were normal with prompt light reflex. Other neurological examinations were normal. He had no lacrimation, nasal running, or conjunctival congestion. There was no skin rash. TN was suspected and he was given acetaminophen, pregabalin, and antihypertensive medication.

**Figure 1:**
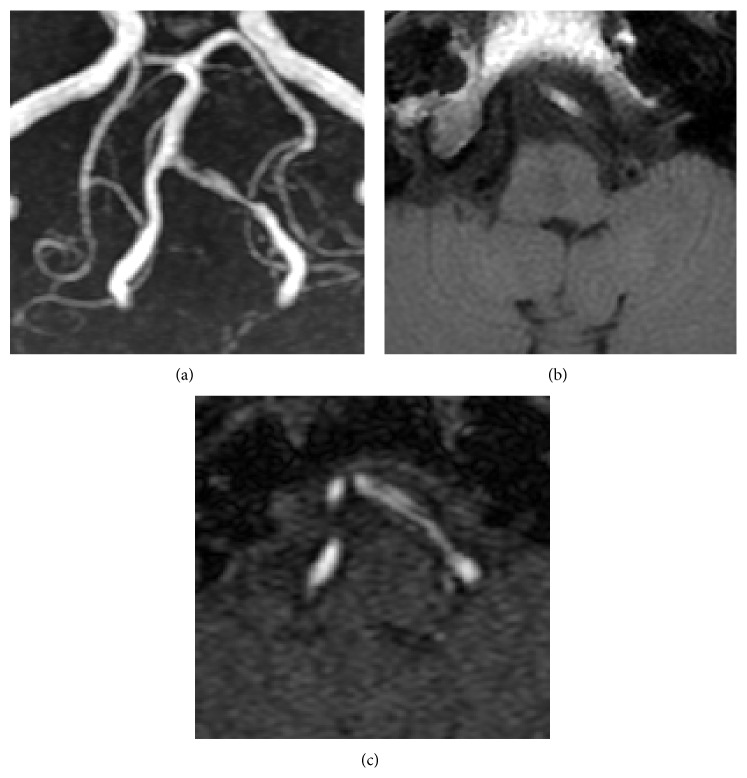
(a) Time-of-flight magnetic resonance angiography (MRA) of the left vertebral artery on admission reveals irregular narrowing of the lumen and a surrounding high-intensity area suggestive of a false lumen or intramural hematoma. (b) The T1-weighted image of MRI demonstrates a high-intensity area in the vessel wall, indicating intramural hematoma. (c) The source image of the MRA demonstrates a linear low-intensity structure presumed to be the intimal flap that separates 2 high-intensity areas.

Magnetic resonance imaging (MRI) of the head was performed to search for possible causes of secondary neuralgia; it did not detect any disease or compression of the trigeminal nerve. However, it revealed irregularity and stenosis of the left vertebral artery with intramural hematoma (Figure 1). The source image of magnetic resonance angiography (MRA) showed the intimal flap. Hence, a diagnosis of IVAD was made.

Ten days later, the spontaneous pain had become less severe, but the patient felt the same brief stabbing pain when his face was touched in the territory of the first division of the trigeminal nerve. A second MRI of the brain revealed further narrowing of the left vertebral artery. He was continued on analgesics.

**Figure 2:**
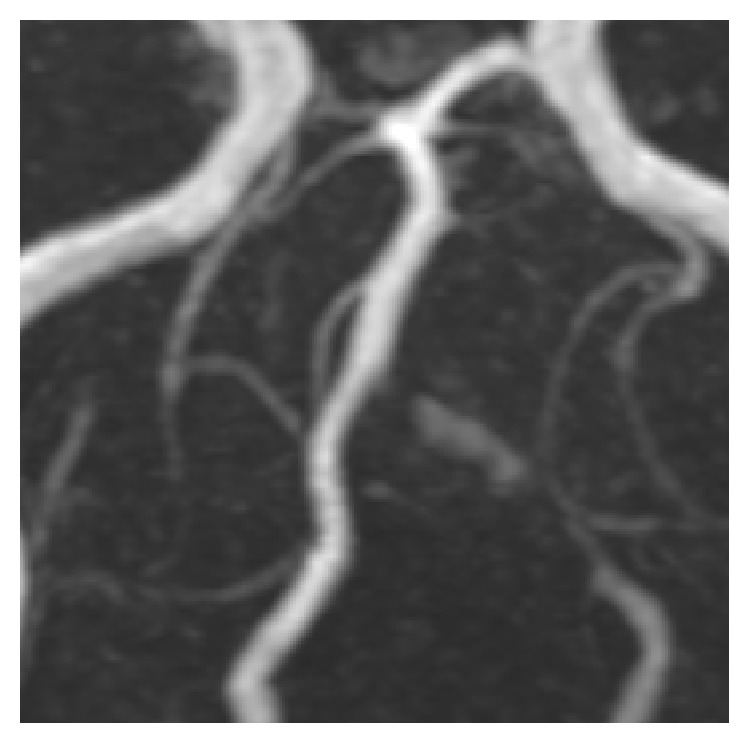
Follow-up MRA performed approximately 6 weeks after the first demonstrates progression of the stenosis of the left vertebral artery. The false lumen or intramural hematoma is now less conspicuous.

Gradually, the facial pain disappeared in approximately 4 weeks. Follow-up MRI showed increased stenosis of the left vertebral artery (Figure 2); the intramural hematoma was less conspicuous than before. The analgesics were then discontinued. Another 3 weeks later, the patient experienced an episode of vertigo lasting for several minutes. A repeat MRI revealed further increase in the degree of stenosis of the left vertebral artery. He was given low-dose aspirin. Thereafter, he remained asymptomatic despite discontinuation of analgesics. Six months later, MRI revealed occlusion of the left vertebral artery. No brain infarction was demonstrated in any of the serial MRIs.

## 3. Discussion

This case indicates that IVAD can present with isolated facial pain. IVAD usually presents with SAH or, more frequently, with ischemic stroke, particularly a lateral medullary infarction [1]. IVAD is often accompanied by headache that is almost always occipital and, infrequently, the only symptom [1, 2]. Facial pain in IVAD is rare; only 9 cases have been reported [3–10]. In eight of them, symptoms and signs of medullary ischemia, such as dizziness/vertigo [4–9], ataxia [6–10], sensory disturbances [7–10], and more [6–10], either preceded [6, 7, 9, 10] or coincided [4, 5, 8] with facial pain; these clinical features helped diagnosis. Furthermore, MRI revealed medullary infarction in these cases, which further facilitated diagnosis. To the best of our knowledge, only 1 case of IVAD has been reported to present with isolated facial pain [3]; in this case, the left facial pain mimicked cluster headache and MRI performed 12 days after onset revealed ipsilateral IVAD without brain infarction. Similar to our case, there was diagnostic difficulty and delay; such scenarios pose some danger because IVAD can cause fatal SAH during its course. Therefore, IVAD should be considered in the differential diagnosis of acute facial pain.

The facial pain in our patient was persistent with a brief stabbing pain triggered by tactile stimuli in the trigeminal nerve territory. These features fulfill the criteria of “classical trigeminal neuralgia (TN) with concomitant persistent facial pain” [11], except that the cause was IVAD. Of the reported cases of facial pain in IVAD, 1 patient suffered from TN-like facial pain 2 days after the onset of IVAD with lateral medullary infarction [10]. However, no case of IVAD presenting with isolated TN-like facial pain has been reported. Our case demonstrates that IVAD can present with isolated TN-like facial pain and suggests that IVAD should be considered in the differential diagnosis of TN.

Although this is the first case published, a similar presentation has been reported in a patient with lateral medullary infarction without IVAD [12]. In that case, the only symptom was persistent and episodic facial pain that was triggered by tactile stimuli. MRI later revealed small infarction in the lateral medulla at the site of the spinothalamic tract; the authors speculated ischemia as the cause of TN-like facial pain. In view of the clinical similarity, although no infarction was demonstrated, ischemia of the spinothalamic tract may have also been involved in our case. In addition, facial pain without TN-like features has been described in other cases of lateral medullary infarction without IVAD [13, 14]. Moreover, Kuwabara and Hirayama [15] reported that “eye-to-forehead headache” was present in 1 of 34 patients with lateral medullary infarction. Therefore, facial pain may be an unusual manifestation of lateral medullary ischemia.

In conclusion, IVAD can present with isolated facial pain that mimics TN. IVAD should be considered in the differential diagnosis of acute facial pain or TN.
